# Unsaturated lipid bodies as a hallmark of inflammation studied by Raman 2D and 3D microscopy

**DOI:** 10.1038/srep40889

**Published:** 2017-01-18

**Authors:** K. Czamara, K. Majzner, A. Selmi, M. Baranska, Y. Ozaki, A. Kaczor

**Affiliations:** 1 Faculty of Chemistry, Jagiellonian University, Ingardena 3, 30-060 Krakow, Poland; 2 Jagiellonian Centre of Experimental Therapeutics (JCET), Jagiellonian University, Bobrzynskiego 14, 30-348 Krakow, Poland; 3 Department of Chemistry, School of Science and Technology, Kwansei Gakuin University, Gakuen 2-1, Sanda, Hyogo 669-1337, Japan

## Abstract

Endothelial HMEC-1 cells incubated with pro-inflammatory cytokine TNF-α for 6 and 24 hours were studied as a model of inflammation using Raman imaging. Striking changes in distribution, composition and concentration of cellular lipids were observed after exposure to TNF-α compared to the control. In particular, 3D Raman imaging revealed a significant increase in the amount of lipid entities formed under inflammation. Lipid bodies were randomly distributed in the cytoplasm and two types of droplets were assembled: more saturated one, in spectral characteristics resembling phosphatidylcholine and saturated cholesteryl esters, observed also in the control, and highly unsaturated one, containing also cholesterols, being a hallmark of inflamed cells. The statistical analysis showed that the number of lipid bodies was significantly dependent on the exposure time to TNF-α. Overall, observed formation of unsaturated lipid droplets can be directly correlated with the increase in production of prostacyclins - endogenous inflammation mediators.

Basic knowledge about the subcellular changes that occur inside the cell body during various cellular events and under disease conditions is extremely important for understanding of the mechanisms of pathology development, increasing the chance of successful diagnostics and treatment. Confocal Raman microscopy is a powerful tool to study cellular model systems due to several reasons: 1. the possibility to obtain comprehensive information about the biochemical composition of the sample, 2. the submicrometer spatial resolution providing information about the changes in the biochemical composition and their location at the subcellular level and, 3. the sensitivity and structural specificity in label-free detection of alterations occurring in organelles and main cellular compartments. All mentioned advantages make Raman microscopy a particularly convenient tool to develop understanding of cellular processes in diseases. One of many potential branches of medicine in which Raman microscopy can be used successfully is cardiology. The growing evidence indicates that some cardiovascular events and lifestyle diseases i.e. atherosclerosis, diabetes and hypertension begin with endothelial dysfunction^1^,^2^,^3^,^4^,^5^ and thereby, endothelial cell cultures are convenient models to study pathology development in the circulatory system. However, only few reports have been previously published regarding human endothelial cells lines cultures studied by means of Raman microscopy^6^,^7^,^8^,^9^,^10^,^11^. So far, it has been applied to characterize the chemical and structural changes related to early apoptosis invoked by exposure to various agents^6^, formation of lipid droplets^7^,^8^ and its role as a cargo in intercellular communication^9^ or to monitor the intracellular pH^10^ and accumulation of bioactive drugs i.e. anthracyclines in studies on mechanisms of endothelial toxicity^11^.

The endothelium is a highly specialized unicellular layer of cells lining the blood and lymph vessels, the natural gatekeeper between the blood and the vascular wall, which is responsible for a wide variety of critical processes controlling vascular functions^1^,^12^. One of the main actions of endothelial cells is associated with triggering of innate and acquired immune response, after activation by e.g. pro-inflammatory cytokines, hypoxia or metabolic stress, in which they produce and release cytokines and growth factors sending signals to leukocytes^13^,^14^,^15^. The action of pathological agents and traditional risk factors including, among others, aging, smoking and hypercholesterolemia, provokes endothelial dysfunction, a process characterized by diminished production or availability of nitric oxide^2^,^16^. Moreover, it is also suggested that the early step of endothelial dysfunction manifests itself in developing of inflammatory states^16^. Inflammation, in general, is intended to limit invasion and damage after the injury, a process that is essential for the survival of organisms. It involves both innate and adaptive immune systems concerning general and specialized line of defense against foreign pathogens, respectively. In the cellular scale, endothelial cells respond to pro-inflammatory factors via several different mechanisms. Activation can occur through specific membrane receptors i.e. the tumor necrosis factor receptor 1 (TNF-R1) by binding of its ligand tumor necrosis factor alpha (TNF-α), the interleukin 1 receptor, type I (IL-1R1) after stimulation by interleukin 1 (IL-1) or toll-like receptor (TLR) when endothelial cells are exposed to endotoxins^13^,^15^. All mentioned here inflammatory mediators activate endothelial cells through the classical nuclear factor kappa-light-chain-enhancer of activated B cells (NF-κB) pathway. NF-κB induces activation of various factors, such as leukocyte adhesion molecules: E-selectin, vascular and intercellular cell adhesion molecule 1 (VCAM-1 and ICAM-1, respectively), and cyclooxygenase-2 (COX-2), required for the inflammatory response^15^. Due to induction of COX-2, endothelial cells exhibit enhanced prostacyclin (PGI_2_) synthesis which is initiated by converting arachidonic acid to prostaglandin H_2_ (PGH_2_)^17^. The presented mechanism of pro-inflammatory factors’ action invokes alterations in lipid compounds characterized by the presence of long non-polar hydrocarbon chains in their structures. Due to large Raman scattering cross-section of lipid signals, bands originating from lipids can be clearly detected in Raman spectra. Thus, Raman microscopy is a great technique to investigate changes in the lipid composition inside complex systems i.e. cells and tissues^8^,^18^,^19^,^20^. Moreover, the possibility to obtain 3D Raman images of cells enables to visualize the distribution and track of newly formed entities inside the cell body. This approach can easily compete with confocal fluorescence imaging where the number of simultaneously analyzed components is limited.

3D Raman imaging is a valuable technique particularly for *in vitro* cellular models and it is being increasingly used in a number of different biological applications. Its advantage primarily lies in the possibility to verify the uptake of various materials by cells i.e. unsaturated fatty acids^7^,^8^, nanoparticles^21^,^22^,^23^ and fibers^24^ and their localization by visualization of subcellular environment. Additionally, 3D profiling enables determination of distribution, size, structure and chemical alterations of cellular components inside the cell^25^,^26^,^27^.

TNF-α is a member of tumor necrosis family, a transmembrane protein mainly expressed by activated monocytes/macrophages and T cells^28^,^29^. Besides, this cytokine is also found in a soluble form in the blood plasma^30^. Both membrane-associated and soluble TNFs possess inflammatory and potent tumoricidal activity^28^,^31^ as well as the ability of inducing apoptosis via activation of caspases^32^. There are scarce reports related to effects of TNF-α stimulation on endothelial cells which are nicely summarized in the recent reviews^31^,^33^. TNF-α may activate a number of signaling pathways in endothelial cells and the main consequences of its action is an increase in the intercellular adhesion molecules secretion and leucocyte adhesion^34^, alterations of nitric oxide expression correlated with elasticity^35^, the increase of reactive oxygen species (ROS) production^33^ and apoptosis^36^. On the other hand, TNF-α is well-known pro-inflammatory agent leading to increased PGI_2_ synthesis which is a marker of alterations in arachidonic acid conversion and confirmation of inflammation. In this work, Raman microscopy and 3D profiling was used for the first time to study the chemically induced inflammation state in endothelial cells in response to TNF-α exposure. We investigated changes of the phenotype of human dermal microvascular endothelial cells (HMEC-1), predominantly related to production of unsaturated lipid droplets. Raman imaging enabled characterization of composition of lipid bodies formed upon inflammation, while 3D profiling showed their exact intercellular distribution. Using 3D imaging, providing insight into the cell, we obtained the ultimate evidence about distribution of cellular components. Lipid droplets may become a universal Raman marker of inflammation enabling testing of new anti-inflammatory drugs in the model systems.

## Results

### Alterations inside endothelial cells after exposure to pro-inflammatory factor TNF-α

**Figure 1:**
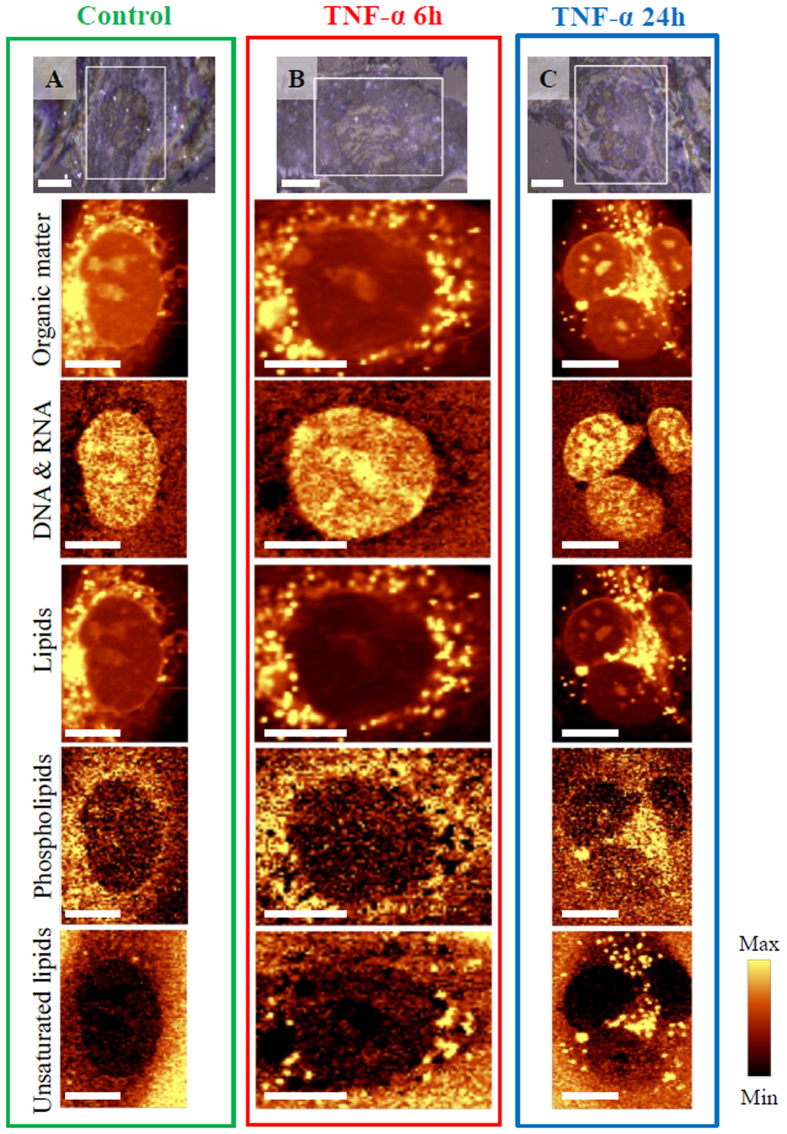
Representative visual and Raman images of HMEC-1 cells: control and incubated with TNF-α for 6 and 24 h. The reflected light microphotographs at 63× magnification (**A–C**) with marked imaged areas and Raman images of distribution obtained by integration in the regions: 2830–3030 cm^−1^ (all organic matter), 760–810 cm^−1^ (DNA & RNA), 2830–2900 cm^−1^ (lipids), 713–733 cm^−1^ (phospholipids) and 3000–3030 cm^−1^ (unsaturated lipid bodies). Scale bars equal 6 μm.

Previously it was demonstrated that, due to its high spatial resolving power, confocal Raman microscopy enabled to visualize the major cell components and determine structural and biochemical alterations in the endothelial cell body after exposure to various factors^6^,^8^,^11^,^37^. In this work, Raman imaging was applied to investigate the influence of TNF-α on biomolecular composition inside HMEC-1 cells. Figure 1 presents representative visual and Raman images showing effects of action of TNF-α on endothelial cells.

The reflected light microphotographs with marked areas of measurements and Raman images of the representative control cell and cells treated with TNF-α for 6 and 24 hours are shown. The two-dimensional Raman images were obtained to visualize the distribution of all organic compounds such as proteins, lipids or nucleic acids: DNA and RNA. Additionally, distribution of phospholipids and unsaturated lipids was selectively determined. The obtained Raman spectra were analyzed by integration of the intensity of the characteristic marker bands assigned to the C–H stretching vibrations (in the region of 2830–3030 cm^−1^), the ring breathing modes in the DNA/RNA bases^38^ at 785 cm^−1^, the C–H stretching vibrations originating mostly from lipids (in the region of 2830–2900 cm^−1^), the symmetric stretching vibrations of the choline N^+^(CH_3_)_3_ groups at 721 cm^−1^ and the stretching modes of =C–H moieties at 3015 cm^−1^, respectively^39^. All bands seen in Raman spectra of cell exposed to TNF-α are listed with their assignments in Table S1 (Table S1, Supplementary Information). The results show that incubation of endothelial HMEC-1 cells with the pro-inflammatory cytokine caused a striking change in the amount and composition of lipid bodies. Lipid bodies are specific organelles composed of a lipid core surrounded by a monolayer of phospholipids with associated proteins^40^,^41^. The formation of lipid bodies is typical for endothelial cells in response to different inflammatory factors as they are sites for the production of inflammatory mediators^41^. Particularly, numerous unsaturated lipid bodies appeared in the cytoplasm after TNF-α stimulation (bottom row, Fig. 1) in respect to the control where these lipid entities were scarce. Moreover, the number of newly formed lipid bodies increased with increasing exposure time (*vide infra*). They were localized only outside the nucleus, suspended in the cytoplasm in the close vicinity to endoplasmic reticulum. TNF-α is a well-known promotor of the inflammatory response and the increase in the number of unsaturated lipids that it caused can be correlated directly with the increase in production of prostacyclins - major endogenous mediators of inflammation^42^. Observed lipid entities were further characterized with the help of depth profiling and cluster analysis.

### Formation and localization of lipid bodies in HMEC-1 cells in inflammation

**Figure 2:**
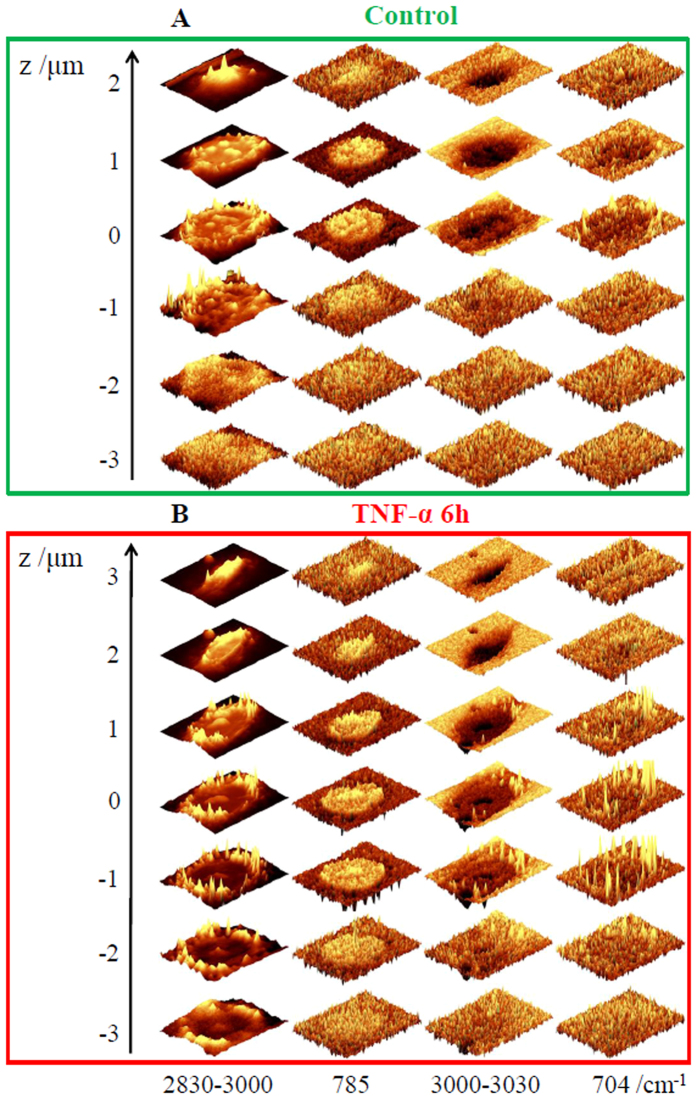
Confocal 3D imaging of control and TNF-α-stimulated HMEC-1 cells. Raman images of distribution for control (**A**) and stimulated with TNF-α (**B**) cells were obtained from layers every 1 μm step in the z-direction by integration in the region of 2830–3030 cm^−1^ (all organic matter), 760–810 cm^−1^ (DNA & RNA), 3000–3030 cm^−1^ (unsaturated lipid bodies) and 695–715 cm^−1^ (cholesterols). Intensities of bands between layers were not normalized.

To investigate distribution of lipid bodies in HMEC-1 cells in inflammation, 3D depth profiling was performed by measuring control and 6 h TNF-α-stimulated cells layer-by-layer every 1.0 μm in the *z*-direction up to down (Fig. 2A and B). Although 3D imaging is in principle achievable in every confocal Raman microscope, it is not often applied in cellular studies as it is both time consuming and requires a microscope of high confocality. Nevertheless, 3D imaging yields clear information about distribution of cellular components and is particularly useful in case of formation of small objects in response to a pathogenic factor, as in this study.

The stacks for representative control/TNF-α-exposed cells confirmed the noteworthy changes in the lipid distribution and composition in cells in inflammation. The analysis of Raman images obtained by integration of marker bands at 3015 and 704 cm^−1^, showed the striking increase in the level of unsaturated lipids and cholesterols^39^, encapsulated in lipid bodies formed in inflamed cells. They occurred in the whole cytoplasm and were randomly distributed, but not present inside the nucleus. The 3D images confirm the intracellular localization of formed lipid entities and gave rise to define their size and shape. The size of observed, almost spherical, lipid droplets in endothelial cells upon development of inflammatory state varied from very small of *ca.* 0.5 μm to larger ones of even 2 μm in diameter. Such variation in size may result from fusion of some smaller droplets into bigger ones. Moreover, 3D images showed unequivocally that unsaturated lipids and cholesterols co-localize in formed lipid structures, suggesting that the produced lipids are cholesteryl esters. The virtually reconstructed 3D Raman image of the cell exposed to TNF-α is given in Fig. S1 (Supplementary Information).

### Raman profile of endothelial cells after exposure to pro-inflammatory factor TNF-α

**Figure 3:**
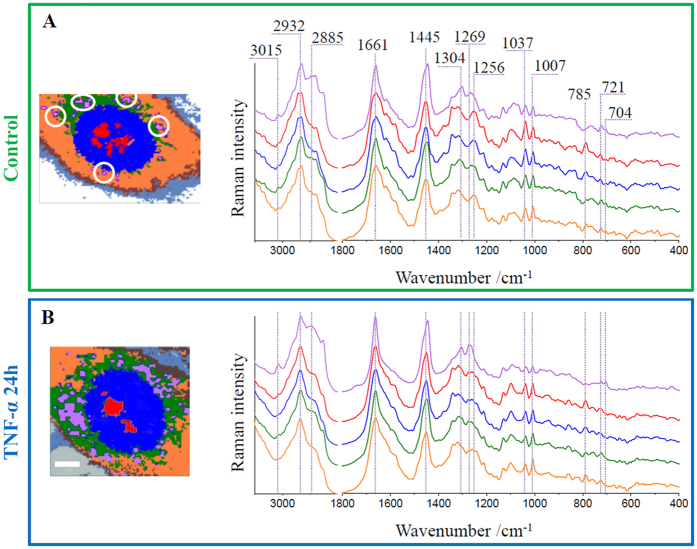
Representative cluster analysis images and average Raman spectra of main classes for control and TNF-α-stimulated HMEC-1 cells. Cluster images of representative control (**A**) and stimulated with TNF-α (**B**) cells (7 classes: purple – lipid bodies, for control marked with white circles, red – nucleoli; blue – nucleus; green – endoplasmic reticulum; orange – cytoplasm, brown – cell membrane; gray – adhesion proteins) and the Raman average spectra of respective main classes. All spectra were maximally extended in the y-axis. Scale bars equal 6 μm.

To extract information about distribution and composition of cellular components and subsequently to define the chemical alterations within main compartments of endothelial cells we used cluster analysis (CA, *k*-means with the Manhattan distance). The results for representative control and TNF-α-exposed HMEC-1 cells are shown in Fig. 3. The applied approach enabled to distinguish main subcellular compartments and organelles i.e. lipid bodies (purple class), nucleoli (red class), nuclei (blue class), endoplasmic reticulum (green class) and cytoplasm including small organelles (orange class), and to spatially separate changes occurring inside the cell and on its surface. For all mentioned classes, the average Raman spectra are presented. Due to low-intensity signal from classes assigned to the cell membrane (brown) and adhesive proteins (gray), they were not included in the analysis.

The striking changes are observed in the class representing lipid droplets. For the control cell there is a small area (marked with white circles) covered by lipid entities, whereas for the TNF-α-stimulated cell a large area of the cell body is occupied by them and their signal is clearly visible. Other classes were not significantly altered after exposure to the pro-inflammatory factor. Raman spectral features of these classes were previously described^6^. In brief, the Raman spectra of nucleoli and nuclei possessed characteristic bands at 785, 1097 and 1580 cm^−1^, attributed to the nucleic acid vibrations of both DNA and RNA molecules^38^. The endoplasmic reticulum class, of a structure composed of membrane lipids, was classified by the presence of the band at 721 cm^−1^ arising from the vibrations of the choline/ethanolamine moieties of phospholipids^39^. The intensity of this band slightly changes in all mentioned classes. Phospholipids, as a basic unit of all membranous structures, are omnipresent inside the cell body. The highest intensity of 721 cm^−1^ band is seen in the endoplasmic reticulum class spectrum, but membranes are also found in cytoplasm as i.e. Golgi apparatus or mitochondria and they surround nuclei and nucleoli as well. In all classes, the band at 1661 cm^−1^ (amide I), the broad band with the maximum at 1255 cm^−1^ (amide III) and couple of features due to phenylalanine at 1007 and 1037 cm^−1^ were observed^43^. The amide III band profile was slightly different for TNF-α-stimulated cells in comparison to control, where the band at *ca.* 1255 cm^−1^ was clearly visible. The origin of this signal can be assigned to the α-helix secondary structure of proteins^38^ and alteration of its intensity indicates the structural transition of proteins. Detailed assignments of bands observed in the average Raman spectra of classes are given in Table S1 (Table S1, Supplementary Information).

**Figure 4:**
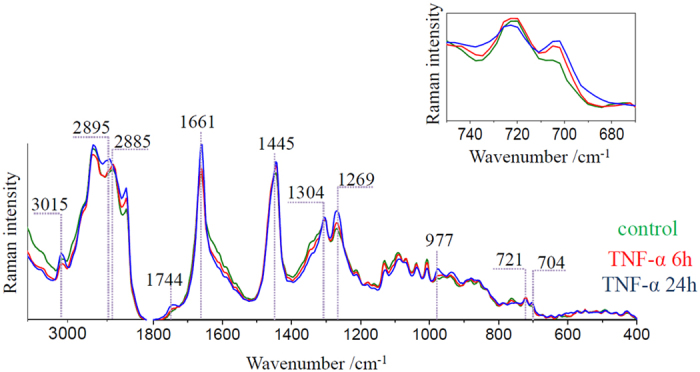
Comparison of Raman spectra of lipid bodies of control and TNF-α-stimulated HMEC-1 cells. Raman spectra of lipid bodies extracted from control (green) and TNF-α-stimulated for 6 (red) and 24 hours (blue) HMEC-1 cells averaged over all measured cells. The insert shows changes in the intensity of the band at 704 cm^−1^. Spectra were normalized in the 1500–400 cm^−1^ spectral range.

The spectra of lipid droplets are unambiguously different from the spectra of other classes. For better analysis of their chemical composition, the spectra of this class were averaged in groups (control, 6 h incubated and 24 h incubated) over all measured cells (at least 7 cells) and presented in Fig. 4. The results reflect typical profiles of lipids with various levels of unsaturation. The fingerprint region shows bands at 977, 1304 and 1445 cm^−1^ originating from the deformations of long hydrocarbon chains: the C-H bending, twisting of the methylene group and the scissoring mode of the CH_2_/CH_3_ groups, respectively^39^. In this region characteristic features of phospholipids and cholesterols are observed at 721 and 704 cm^−1^, respectively (see the insert in Fig. 4). The unsaturation reveals in the spectrum as the presence of bands at 3015, 1661 and 1269 cm^−1^, assigned to the =C–H stretching, C = C stretching and in-plane C–H bending modes, respectively. The degree of unsaturation can be determined as the n(C = C)/n(CH_2_) intensity ratio obtained by integration of the respective Raman bands (1655/1444 cm^–1^) and fitted to a calibration curve for unsaturated fatty acids (Fig. S2, Supplementary Information)^8^,^39^. For average spectra of lipid droplets from cells exposed to TNF-α for 6 and 24 hours the ratio equals 2.28 and 2.33, respectively. Additionally, the low-intensity band associated with the stretching vibration of the C = O group in cholesteryl esters^39^ is observed at 1744 cm^−1^. The presence of bands due to various lipid functional groups indicates the mixture of lipids inside the lipid bodies. Moreover, the comparison of intensity ratios of marker bands between analyzed groups shows that changes are dependent on the incubation time with TNF-α. The longer exposure to the pro-inflammatory factor causes the elevation in the level of unsaturated lipids observed as the increase of bands at 1661 and 1269 cm^−1^. The similar, but less evident, effect is observed for bands at 704 and 1744 cm^−1^ related to cholesteryl esters. The increase in unsaturated lipids content upon inflammation is also manifested in the high spectral range. The band due to the asymmetric stretching vibration is observed at 2885 cm^−1^ for the control while for lipid droplets after 24 hours of stimulation it is shifted to the higher wavenumber i.e. 2895 cm^−1^, a wavenumber characteristic for lipids with double bonds in their structure^39^.

### Heterogeneity of lipid bodies under the progress of inflammation

**Figure 5:**
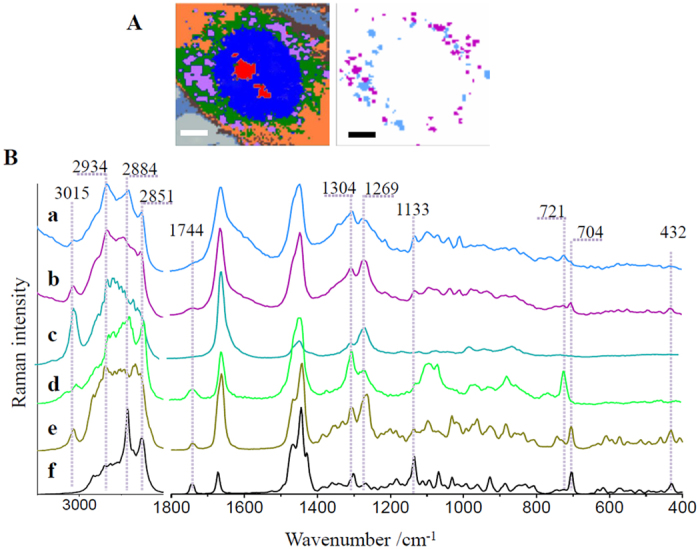
Heterogeneity of lipid bodies. Representative cluster analysis (**A**) for a HMEC-1 cell exposed to TNF-α and subclasses of lipid bodies with their respective Raman spectra (a and b) compared to Raman spectra of analytical standards of lipids: arachidonic acid (c), phosphatidylcholine (d), cholesteryl arachidonate (e) and cholesteryl stearate (f). All spectra were maximally extended in the y-axis. Scale bars equal 6 μm.

Numerous features originating from different groups of lipids in Raman spectra of lipid droplets indicate heterogeneity in their composition. To account for the complexity of lipid droplets, the extracted lipid droplet classes were subsequently divided into subclasses. Analysis of Raman spectra enabled to separate two classes with significantly different Raman profiles (denoted by cyan and violet in Fig. 5A, other classes as in Fig. 3) representing lipid bodies of a considerably distinctive chemical composition (compare Fig. S3, Supplementary Information).

To verify that fixation does not affect lipids, especially phospholipids with the amine group, an additional experiment was performed showing that the effect of glutaraldehyde on lipids (in this case) is irrelevant (Fig. S4, Supplementary Information). Raman spectra of lipid droplets subclasses (Fig. 5B a and b) are shown together with Raman spectra of analytical standards of lipids (Fig. 5B c–f). The comparison of spectral profiles reveals that lipid bodies represented by the cyan subclass are composed of phospholipids as indicated, among others, by the presence of the marker band of choline at 721 cm^−1^. Moreover, lipid droplets in this class possess more saturated characteristics. In the high frequency region bands at 2851 and 2884 cm^−1^ originating from the C-H stretching vibrations in fatty acid chains have comparable intensity to these bands in the spectra of phosphatidylcholine (d) and cholesteryl stearate (f). Contrarily, the intensity of bands at 3015 and 1269 cm^−1^ assigned to the =C–H stretching and in-plane C–H bending modes, respectively, are of lower intensity. The features typical for sterol ring deformations of cholesterols at 432 and 704 cm^−1^ are observed in the spectrum of this class, although their intensity is significantly lower compared to the intensity of these signals in the second type of lipid bodies (violet subclass), composed mainly of unsaturated lipids and cholesterols. The detailed comparison of spectra of pure cholesterols and a spectrum of unsaturated lipid droplets in the 800–400 cm^−1^ spectral range enables to clearly confirm that cholesterol esters rather than unbound cholesterol are components of droplets (Fig. S5, Supplementary Information). The differences are seen in the position of the band at *ca.* 430 cm^−1^ (424 and 432 cm^−1^ for cholesterol and a cholesteryl arachidonate, respectively) as well as in the intensity ratio of bands at 704, 548 and about 430 cm^−1^. Thus, the Raman signature of unsaturated lipid droplets resembles the profile of cholesteryl arachidonate in agreement with the fact that cholesteryl esters can occur in these structures^40^.

**Figure 6:**
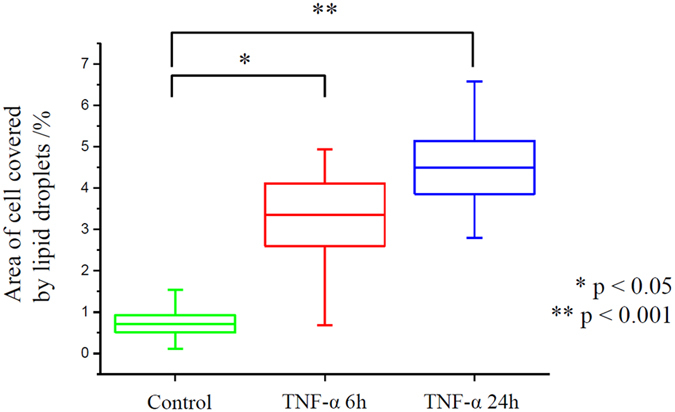
Size statistics of lipid bodies upon time dependent exposure to TNF-α. The calculated area of lipid bodies is referred to the total cell surface.

Together with the identification of different lipids in the structures of lipid bodies, the area of Raman images covered by them was calculated with respect to the whole cell (Fig. 6).

This analysis was performed on Raman lipid droplets classes extracted using CA. The results show that the incubation with TNF-α leads to statistically significant changes in the total number of lipid entities (p values, with 5% level of decision using the Tukey’s test in ANOVA, equal to 0.015 and 0.0009 for 6 h and 24 h incubation, respectively) calculated as the percent of the area of the whole cell covered by the lipid bodies. It also reveals that progression of inflammation was dependent on the incubation time. However, for the longer time of exposure the apoptotic properties of TNF-α might have essential impact on cell viability and could dominate over pro-inflammatory effect^36^. The increase of the overall size of the surface covered with lipid bodies caused by formation of droplets of unsaturated character is characteristic for inflammation.

## Discussion

It is known that lipid bodies can be rapidly formed in resting leukocytes under the influence of adequate stimuli^41^. *In vivo* lipid bodies biogenesis occurs in response to inflammation, among others in macrophages in atherosclerotic lesions, eosinophils in allergic inflammation or leukocytes in bacterial sepsis. These and other examples of lipid bodies synthesis in response to inflammation are reviewed thoroughly in the report by Melo *et al*.^41^.

In our cellular model of endothelial inflammation, i.e. HMEC-1 cells exposed to TNF-α, we observed formation of lipid droplets containing lipids of unsaturated characteristics. The lipid bodies formation was dependent on the incubation time and significantly increased with a longer exposure time to the pro-inflammatory factor. The formed lipid droplets, spanning the size of *ca.* 0.5–2 μm, were distributed in the cytoplasm of the cells exposed to TNF-α.

Interestingly, the lipid entities observed in the inflamed endothelial cells were of two subtypes: more saturated, rich in phosphatidylcholine and saturated cholesteryl esters and unsaturated, containing mostly unsaturated lipids and cholesterols. 3D images brought unequivocal confirmation that observed lipid bodies were localized in the cytoplasm inside the cell, but also enabled for a clear co-localization of distribution of formed unsaturated lipids and cholesterols, suggesting that the produced lipids are cholesteryl esters. It is known that lipid droplets occur normally in most cells so it seems that the first subtype represents lipid droplets involved in lipid metabolism and storage^44^,^45^. Droplets of unsaturated characteristics are formed in pathologies and are related to prostaglandin synthesis^46^,^47^. It is known that lipid bodies within inflammatory cells contain arachidonyl lipids^41^. The rate limiting step of eicosanoid production is release of free arachidonic acid hydrolyzed from membrane phospholipids by phospholipases^48^,^49^. Then, free arachidonic acid is rapidly converted into oxygenated products, among others *via* cyclooxygenase pathway, into prostaglandins^48^,^49^. This effect is not commonly described for endothelial cells^50^, probably due to the problem with detection of unsaturated lipids inside the cell body. Our results, based on Raman microscopy, that is a perfect technique to investigate lipid changes, confirm this action of TNF-α. Overall, observed changes show that development of inflammation manifests itself by the elevated concentration of unsaturated lipids that is related to the increase in production of endogenous mediators of inflammation – prostacyclins, highly unsaturated lipids^41^,^42^. The unsaturated lipid bodies, observed in the cells stimulated with TNF-α have a Raman signature resembling the Raman signature of arachidonic acid and arachidonates, therefore they are undoubtedly related to the eicosanoid synthesis pathway.

In the light of our observation, it is shown that applied model i.e. incubation of HMEC-1 cells with TNF-α is a reliable model of inflammation while used methodology (Raman (3D) imaging with chemometric data analysis) enables detailed, label-free characterization of lipid bodies, synthesized in endothelial cells in reaction to inflammation. Our study shows that endothelial inflammation results in distinctive changes in the cells, easily observed in Raman images. This result may be considerably interesting from the point of future studies, particularly application of cellular models in testing of new anti-inflammatory drugs.

## Methods

### Cells culture

A HMEC-1 cell line of human dermal microvascular endothelial cells was chosen to investigate *in vitro* model of inflammation of endothelium. HMEC-1 cells are immortalized cells obtained by transfection of native HMEC cells with a pSVT DNA vector, a PBR-322 based plasmid containing the coding region of the simian virus 40 A gene product, i.e. a large T antigen, to overcome the effect of cell senescence^51^. The cells for Raman measurements were directly seeded onto uncoated CaF_2_ slides (25 × 2 mm, Crystran LTD, UK) at a concentration of 2·10^5^ cells per well to achieve the confluence at the level of about 70%. Cells were cultured in complete MCDB131 medium (Gibco Life Technologies) supplemented with 10 mM L-glutamine (Gibco Life Technologies), 1 μg·mL^−1^ hydrocortisone (Sigma Aldrich), 10 mg·mL^−1^ epidermal growth factor (EGF, Sigma Aldrich), 10% fetal bovine serum (FBS, Gibco Life Technologies) and antibiotic antimycotic solution (AAS with 10.000 U penicillin, 10 mg streptomycin and 25 μg amphotericin B per mL) and maintained at 37 °C in atmosphere of air with 5% CO_2_ in a cell culture incubator. After 24 hours of incubation, enabling the attachment of all cells to the slide surface, they were rinsed twice with phosphate buffered saline (PBS, pH 7.4, Gibco Life Technologies) and exposed to 10 ng·mL^−1^ tumor necrosis factor alpha (TNF-α, Sigma Aldrich) dissolved in fresh medium for 6 or 24 hours, respectively. The incubation times and doses of the TNF-α were estimated based on previous reports^35^ and to avoid its apoptotic effect on cells^36^. Untreated HMEC-1 cells maintained in the medium were used as a control. After stimulation cells were washed twice with PBS and fixed with 2.5% solution of glutaraldehyde in PBS for 4 minutes. The samples were stored in the PBS buffer at 4 °C until Raman measurements.

### Raman microscopy

Raman imaging was carried out using a WITec Confocal Raman Imaging system (WITec alpha300, Ulm, Germany) supplied with a UHTS 300 spectrograph (600 grooves·mm^−1^ grating, resolution of 3 cm^−1^) and a CCD detector (Andor, DU401A-BV-352). The air-cooled solid state laser with the excitation wavelength of 532 nm was coupled to the microscope *via* an optical fiber with a diameter of 50 μm. Raman spectra of endothelial cells were collected with the application of a 63× water immersive objective (Zeiss Fluor, NA = 1.0) and using maximum laser power at the sample position (*ca.* 30 mW). For each cell a 0.7 second exposure time per spectrum was applied. The nominal minimal lateral and depth resolution for our setup is 0.32 and 0.53 μm, respectively, and sampling density of 0.3 and 1 μm in *x*/*y* and *z* direction, respectively, was used. Imaged areas of at least 5 cells in two independent experiments for each group (control, TNF-α in 6 and 24 h exposure time) were measured. 3D experiments were performed by measuring repeatedly the area of the entire cells changing the focal distance (5–7 layers in 1 μm step in *z*-axis). The distribution images collected at different depths present the relative intensity of a studied component in the cell.

Raman spectra of lipid standards were obtained after placement of a standard sample on CaF_2_ slides with the application of the 100× air objective (Olympus MPLAN, NA = 0.90) and the analogous WITec system. Each spectrum was excited with maximum *ca.* 20 mW laser power at sample position with 100 scans and integration time of 0.5 s.

### Data analysis and processing

Data matrices were preprocessed using the WITec Project Plus software. Raman spectra were baseline corrected using autopolynomial of degree 3 and were submitted to an automatic cosmic rays removal procedure. The cluster analysis (CA) was performed with *k*-means method using the Manhattan distance (WITec Project Plus). This approach enabled data grouping into classes and extraction of average spectra reflecting the major organelles within cells. The calculations of areas covered by lipid droplets were done with ImageJ processing program^52^. Results were tested by analysis of variance performed in the OriginPro 9.1 software (ANOVA model with Tukey’s test) to characterize quantitatively the differences in size statistics of the lipid bodies content in all pairwise comparisons for each group (control and incubated with TNF-α for 6 h and 24 h).

## Additional Information

**How to cite this article**: Czamara, K. *et al*. Unsaturated lipid bodies as a hallmark of inflammation studied by Raman 2D and 3D microscopy. *Sci. Rep.* **7**, 40889; doi: 10.1038/srep40889 (2017).

**Publisher's note:** Springer Nature remains neutral with regard to jurisdictional claims in published maps and institutional affiliations.

## Supplementary Material

Supplementary Information
