# Supplementary material for: Unsaturated lipid bodies as a hallmark of inflammation studied by Raman 2D and 3D microscopy
Source: Sci Rep. 2017 Jan 18;7:40889. doi: 10.1038/srep40889 (PMC5241649; doi:10.1038/srep40889)
Supplement: Supplementary Information [file srep40889-s1.pdf]

Supplementary Information

Unsaturated lipid bodies as a hallmark of inflammation studied by Raman 2D and 3D microscopy

K. Czamara[1,2], K. Majzner[1,2], A. Selmi[2], M. Baranska[1,2], Y. Ozaki[3], A. Kaczor[1,2,*]

[1]*Faculty of Chemistry, Jagiellonian University, Ingardena 3, 30-060 Krakow, Poland.*

[2]*Jagiellonian Centre of Experimental Therapeutics (JCET), Jagiellonian University, Bobrzynskiego 14, 30-348 Krakow, Poland.*

[3]*Department of Chemistry, School of Science and Technology, Kwansei Gakuin University, Gakuen 2-1, Sanda, Hyogo 669-1337, Japan*

Table S1. Band assignments for HMEC-1 endothelial cells exposed to TNF-α.

Band position /cm^{-1}	Assignment	References
3015	ν(=C–H)	1
2932	$\nu_{as}(CH_3)$	1
2885	$\nu_{as}(CH_2)$	1
1661	ν(C=C) / amide I	1,2
1445	$\alpha(CH_2/CH_3)$	1
1304	$\tau(CH_2)$	1
1269	δ(=CH)	
1255	amide III	2
1037	Phe	2
1007	Phe	2
785	ring breathing modes in DNA/RNA bases	3
721	$\nu(N^+(CH_3)_3)$ of choline	1
704	ring deformation of cholesterol rings	1

ν – stretching, ω – wagging, δ – deformation, τ – twisting; Phe - phenylalanine

1. Czamara, K. *et al.* Raman spectroscopy of lipids: a review. *J. Raman Spectrosc.* **46,** 4–20 (2015).

2. Rygula, A. *et al.* Raman spectroscopy of proteins: a review. *J. Raman Spectrosc.* **44,** 1061–1076 (2013).

3. Movasaghi, Z., Rehman, S. & Rehman, I. U. Raman Spectroscopy of Biological Tissues. *Appl. Spectrosc. Rev.* **42,** 493–541 (2007).

The ImageJ equipped with Volume Viewer application was used to visualize the 3-dimensional distribution of lipid bodies within cells.

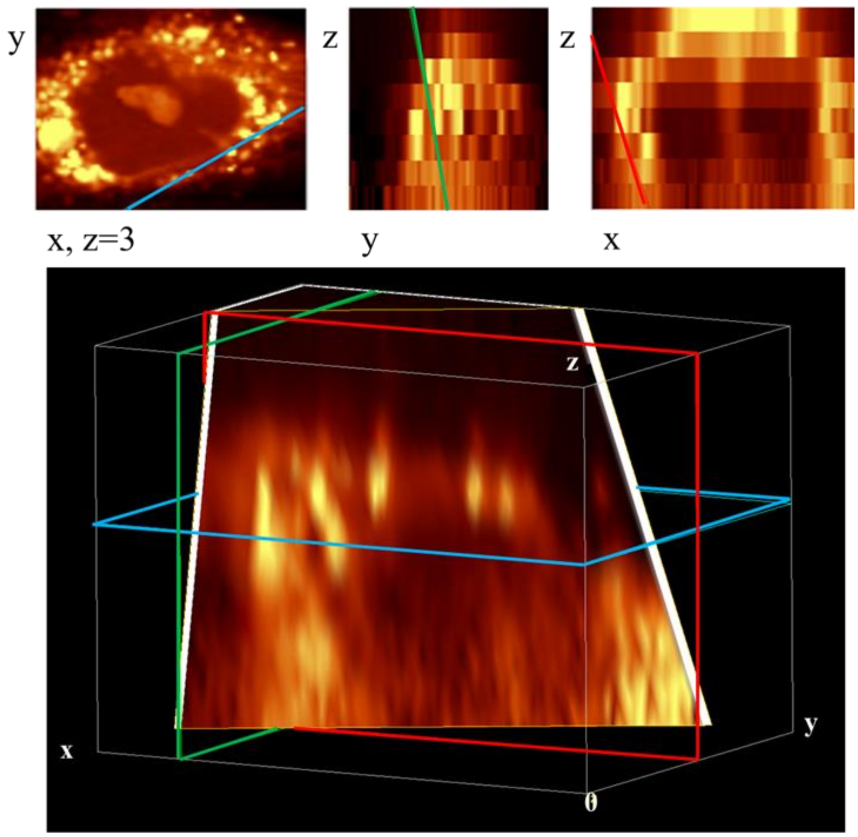

Figure S1. Confocal 3D imaging of control and TNF-α-stimulated HMEC-1 cells. The cross-section of the reconstructed 3D image of a representative cell exposed to TNF-α for 6 h.

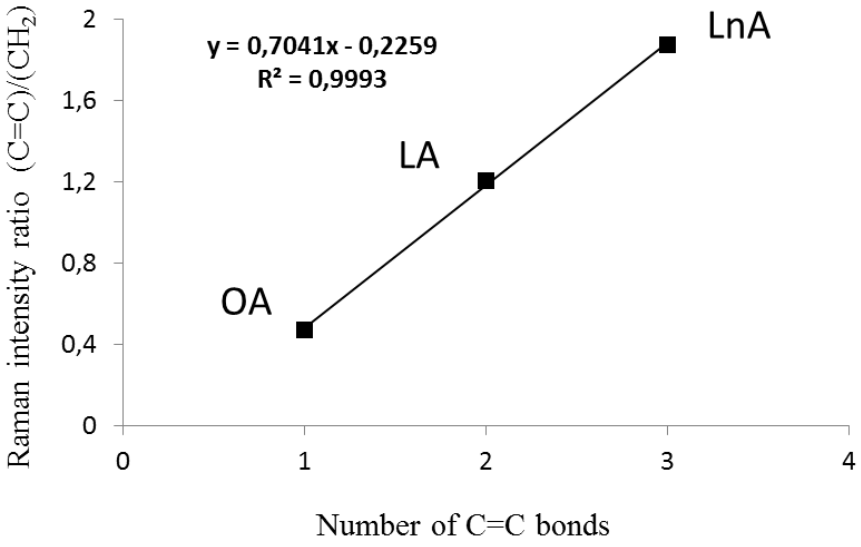

Figure S2. Calibration curve for estimated degree of unsaturation. The calibration curve calculated for selected standards of fatty acids (OA – oleic acid, LA – linoleic acid, LnA – linolenic acid), which correlates their Raman intensity ratio of (C=C)/(CH_2) modes (1660/1444 cm^{-1}) with a number of C=C bonds.

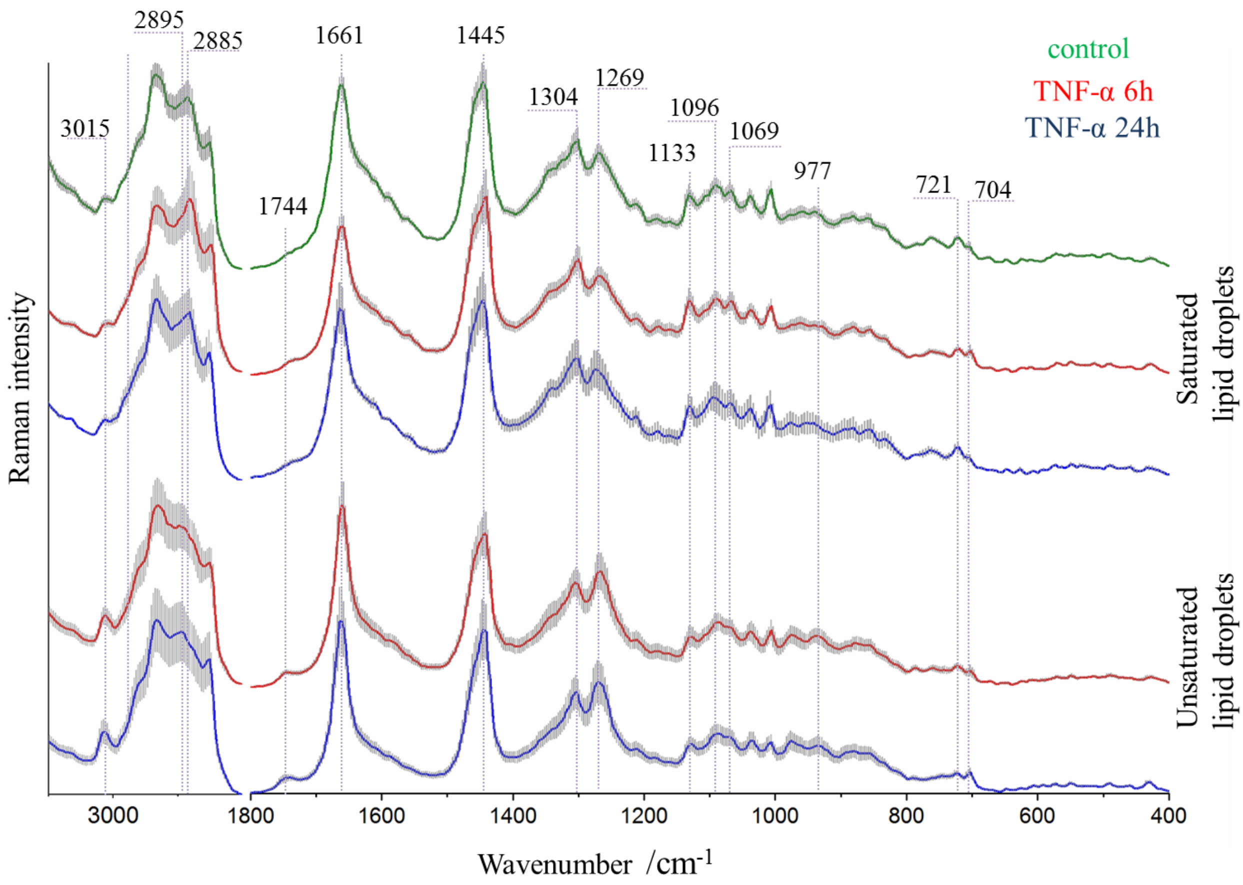

Figure S3. Average Raman spectra of two types of lipid droplets of control and TNF-α-stimulated HMEC-1 cells. Raman spectra extracted from subclass of lipid droplets from control (green) and exposed to TNF-α for 6 and 24 h (red and blue, respectively) averaged and given with standard deviation on each data point.

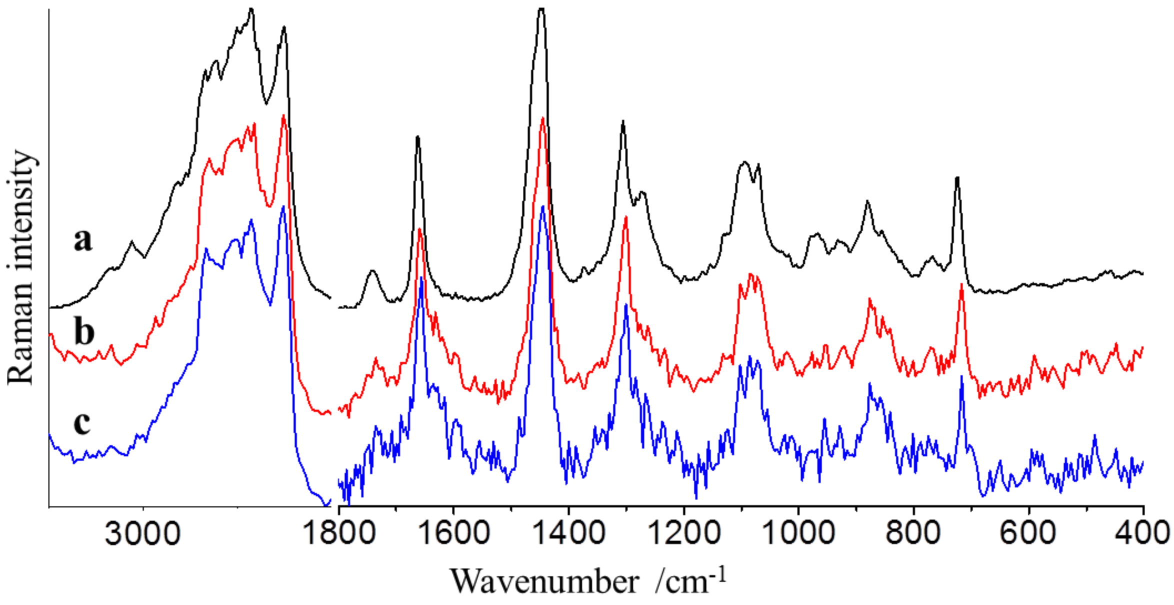

Figure S4. Effect of glutaraldehyde fixation on phospholipids. The comparison of Raman spectra of phosphatidylcholine measured in the solid state (a), after exposure to glutaraldehyde (2,5%, 4 min) in PBS (b) and without exposure in PBS buffer (c).

Fig. S4 shows that the position of bands in both (b) and (c) Raman spectra is the same with no visible alterations in Raman features due to exposure to glutaraldehyde. Additionally, the comparison with the spectrum of phosphatidylcholine in the solid state (a) reveals that dissolving of glutaraldehyde does not significantly change the spectrum. Thus, the effect of glutaraldehyde on lipids (in this case) is irrelevant and comparing of spectra of lipid standards recorded in the solid state with spectra extracted from fixed cells is justified.

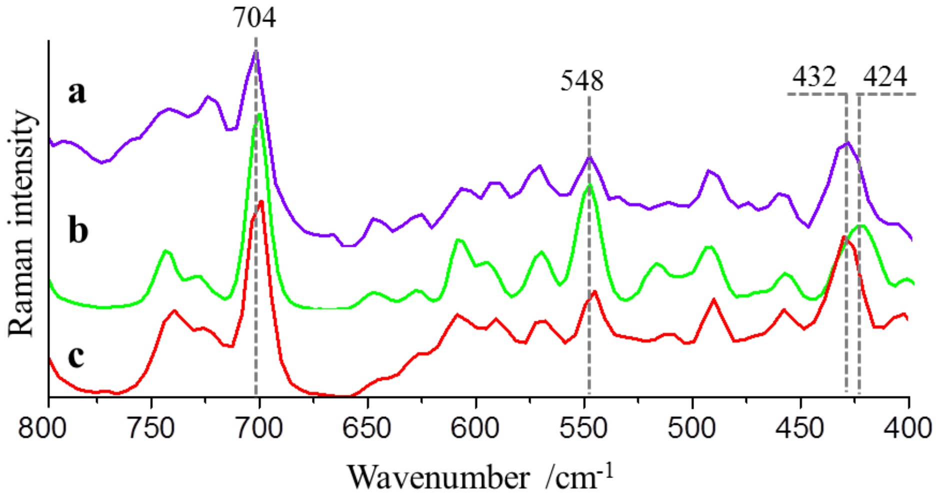

Figure S5. Comparison of Raman spectrum of unsaturated lipid bodies with cholesterols. and cholesteryl arachidonate. Raman spectrum of lipid droplet subclass (a) compared to Raman spectra of analytical standards of cholesterol (b) and cholesteryl arachidonate (e) in 800 – 400 cm^{-1} spectral range. All spectra were maximally extended in the y-axis.

Fig. S5 presents Raman spectra of lipid droplet class, cholesterol and cholesteryl arachidonate enlarged in the 800-400 cm^{-1} spectral region. Here, we can distinguish characteristic bands originating from sterol rings deformations. The differences between pure lipids are seen in the position of the band at about 430 cm^{-1} (424 and 432 cm^{-1} for cholesterol and a cholesteryl arachidonate, respectively) as well as in the intensity ratio of bands at 704, 548 and about 430 cm^{-1}. Thus, the Raman spectrum of the lipid droplets resembles the spectrum of cholesteryl arachidonate; the band at 548 cm^{-1} is significantly less intense relatively to the band at ca. 430 cm^{-1}. Moreover, the latter band in the spectrum of unsaturated lipid droplets is observed at 432 cm^{-1}, as in the spectrum of cholesteryl arachidonate. Such a position of this band is characteristic for all cholesteryl esters.
